# A new three‐dimensional model to describe human corneal oxygenation during contact lenses wear

**DOI:** 10.1111/opo.13510

**Published:** 2025-04-19

**Authors:** José M. Gozálvez‐Zafrilla, Marcel Aguilella‐Arzo, Vicente Compañ

**Affiliations:** ^1^ Institute for Industrial, Radiophysical and Environmental Safety (ISIRYM), Departamento de Ingeniería Química y Nuclear Universitat Politécnica de Valencia Campus de Vera Valencia Spain; ^2^ Departamento de Física Universitat Jaume I Castellón Spain; ^3^ Departamento de Termodinámica Aplicada, Escuela Técnica Superior de Ingeniería Industrial (ETSII) Universitat Politècnica de Valencia Campus de Vera Valencia Spain

**Keywords:** 3‐D model, corneal oxygen consumption, optical power, oxygen flux, oxygen tension, soft contact lens

## Abstract

**Purpose:**

This three‐dimensional study investigated how different contact lens materials affect oxygen levels in the cornea. Specifically, it measured oxygen tension, flux and consumption in the epithelium, stroma and endothelium when exposed to various contact lenses. The goal was to understand how oxygen distribution within the cornea changes based on the oxygen tension at the cornea–tear interface, which is influenced by the lens's oxygen transmissibility.

**Methods:**

To achieve this goal, a finite element analysis model was used that accounted for the axisymmetric properties of the cornea. A parametric analysis was conducted to examine how lens power and refractive index impacted oxygen distribution. This involved testing various contact lens materials with different powers (±3 and ±6D) and refractive indices.

**Results:**

This three‐dimensional model provides new insights into the flux and concentration profiles of oxygen across the epithelium, stroma and endothelium for contact lenses having different optical powers. The key findings show that contact lens thickness and refractive index, which are related to the power, significantly impact oxygen concentration within the cornea. Notably, reduced corneal oxygen consumption occurs primarily at the epithelium, where oxygen tension decreases under both open‐eye and closed‐eye conditions. This decrease depends on the oxygen permeability of the contact lens being worn and its power.

**Conclusions:**

The cornea can sustain normal metabolic processes (aerobic metabolism) if the oxygen levels at the cornea–tear film interface are within approximately 60–100 mmHg. This holds true for all of the contact lenses tested here under open‐eye conditions. However, when the eyes are closed, the cornea is unable to maintain normal metabolic processes, leading to a shift towards anaerobic metabolism. Prolonged exposure to these conditions can cause corneal oedema (swelling) due to an inadequate oxygen supply.

**Key points**

- A three‐dimensional study quantified corneal oxygen consumption using a finite element analysis model considering axis‐symmetric geometry.
- Parametric studies of the power effect for three different contact lens materials were performed under open and closed‐eye conditions.
- This novel model calculates oxygen tension profiles from the central‐to‐peripheral cornea, allowing for the quantification of metabolite transport and assessment of corneal oedema.

## INTRODUCTION

Understanding the oxygen demand of the cornea when using contact lenses has received significant attention in recent years, and numerous models have been developed.^1^, ^2^, ^3^, ^4^, ^5^, ^6^, ^7^ Some have assumed one‐dimensional (1‐D) geometry for oxygen transport through the cornea–tear–lens system, based on the earlier works of Fatt and co‐workers.^4^, ^5^, ^6^ These models typically assume a constant thickness and constant oxygen consumption rate for the multilayer system, treating the diffusion equation only under steady‐state conditions.^2^, ^3^, ^4^, ^5^, ^6^, ^7^ However, such assumptions lead to negative partial pressure values within the cornea, which are physically unrealistic. To address this issue, recent mathematical models have focused on determining the oxygen tension at the cornea–tear interface, where the oxygen consumption rate is considered to be a function of pressure using the nonlinear Monod kinetics model.^8^, ^9^, ^10^, ^11^, ^12^, ^13^, ^14^, ^15^, ^16^, ^17^, ^18^, ^19^, ^20^, ^21^, ^22^, ^23^, ^24^ These models rely on knowledge of the partial pressure of oxygen at the cornea–tear interface, as previously determined by Bonanno et al. with a time‐domain phosphorimeter,^9^, ^17^, ^18^, ^19^, ^21^ with some modifications.^9^, ^17^, ^18^, ^19^, ^21^ However, most of these models are limited to 1‐D representations to try and explain the distribution of oxygen flux and consumption across different regions of the cornea, namely the epithelium, stroma and endothelium.^6^, ^7^, ^8^, ^9^, ^10^, ^11^, ^12^, ^13^, ^14^, ^15^, ^16^, ^17^, ^18^, ^19^

In recent years, researchers have attempted to develop more realistic models by expanding to two‐dimensional (2‐D) and three‐dimensional (3‐D) representations.^20^, ^21^, ^22^, ^23^, ^24^, ^25^ For example, Alvord et al.^20^ incorporated realistic geometry of the cornea and lens system using a 2‐D axisymmetric finite element analysis (FEA) model to study corneal oxygen distribution with a −3.00D contact lens. Their findings demonstrated the significance of lateral thickness, showing that 1‐D models underestimated the oxygen demand across the entire cornea due to its increased thickness in the periphery. Thus, considering lateral thickness variations becomes crucial when studying oxygen transport through the complete cornea, particularly with thick lenses such as scleral contact lenses.

More recently, Takatori et al.^23^ and Kim et al.^21^ introduced ‘quasi‐2‐D’ models that incorporated metabolic reactions involving carbon dioxide, glucose, lactate and other substances within the corneal tissue. These models divided the cornea–tear–lens system into a series of truncated, concentric spherical sectors, effectively converting the 3‐D diffusion into an axisymmetric system that approximated a 1‐D study. However, this approach disregarded oxygen flow in the radial direction, limiting the incorporation of potential oxygen flow from the peripheral cornea, as demonstrated by Alvord et al.,^20^ Kim et al.^21^ and Takatori and Radke.^23^

The advantage of the models proposed by Takatori et al.^19^, ^23^ and Kim^21^ over that of Alvord^20^ lies in their incorporation of diffusion and metabolic reactions, such as carbon dioxide, glucose, lactate, bicarbonate and hydrogen ions within the cornea. However, Compañ et al.^24^, ^25^ developed a model that improves upon the works of Takatori et al.^21^ and Kim.^23^ Compañ et al. provide a comprehensive 3‐D treatment with axial symmetry, which facilitates incorporation of the flux of other metabolites such as carbon dioxide, glucose, lactate ion, bicarbonate ion, hydrogen ion, sodium ion and chloride ion. Moreover, it allows studying a wide range of lenses by constructing a six‐layered system (endothelium–stroma–epithelium/tear/lens/tear) in real time using a few parameters. This model accounts for the maximum consumption rate, which occurs in the epithelium, and is dependent on the oxygen tension at the cornea–tear–lens interface.^24^, ^25^, ^26^ However, our previous models had limitations since they assumed the cornea was subject to a specific contact lens with a given transmissibility and power (−3.00 D). This restricts their applicability to other geometries determined by the power of different contact lens materials.

The aim of the present study was to enhance previously published works,^24^, ^25^, ^26^ where a 3‐D treatment with axial symmetry was implemented considering that the maximum oxygen consumption rate is focused on the epithelium, being a function of the corneal oxygen tension at the cornea/tear/lens interface.^25^ A rigorous and comprehensive model was developed to simulate oxygen diffusion in the cornea–tear–lens system. This model considers various factors that affect oxygen transport, including the lens's transmissibility, geometry and power. To achieve this, 3‐D diffusion equations were solved with axial symmetry, incorporating oxygen flux from both the atmosphere (through the peripheral corneal region) and aqueous humour.

The model is designed to accommodate contact lenses made of different materials with varying powers, ranging from ±3.00 to ±6.00 D. Furthermore, the study analysed how changes in the refractive index impact oxygen diffusion under both open‐ and closed‐eye conditions. The model requires minimal input parameters, making it an efficient tool for simulating diverse lens scenarios. By solving the transport equations numerically, a wide range of output values was obtained, including oxygen pressure profiles, oxygen flows, oxygen consumption rates and integrated values across different segments of the cornea, such as the epithelium, stroma and endothelium. This comprehensive approach allows for a detailed understanding of oxygen diffusion in the cornea–tear–lens system, providing valuable insights for contact lens design and development.

Furthermore, this new model calculates oxygen tension profiles from the central‐to‐peripheral cornea and can quantify metabolite transport to determine corneal oedema. This enables the assessment of the effects of metabolite support from the vascularised limbus and the higher metabolic demand of the mid‐peripheral and peripheral cornea during soft contact lens wear on corneal oedema. In contrast, Alvord's model^20^ cannot account for the effect of metabolite support from the limbus, while Takatori and Radke's model was not able to assess radial metabolite flow, which may be significant depending on the geometry and lens parameters.^23^ By incorporating metabolic support from the limbus and considering various transport mechanisms, the current model offers a new tool to predict the oxygenation safety of contact lens wear throughout the entire cornea.

## MATERIALS AND METHODS

### Lens parameters

Three contact lenses (CLs) were selected: one hydrogel (Hy): Galyfilcon A and two Siloxane‐Hydrogels (Si‐Hy): Balafilcon A and Lotrafilcon A. The characteristics of the lens, water content, central thickness, oxygen permeability and transmissibility are shown in Table 1. Technical details of the CLs, as reported by the manufacturer, are also provided with asterisks in Table 1. The oxygen tension at the cornea/tear/lens interface for the analysed lenses has been obtained previously by Bonanno et al.^9^, ^17^ using the phosphorescence decay methodology.

**TABLE 1:** Physical parameters of the lens materials used in this study.

Lens material	Manufacturer	Water uptake content (%)	Central thickness (μm)	Permeability (Barrer)	Transmissibility (hBarrer/cm)
Galyfilcon A	Johnson & Johnson	47	71	59.4 (60*)	83.7 (85*)
Balafilcon A	Bausch & Lomb	36	100	100.5 (99*)	100.0 (99*)
Lotrafilcon A	Alcon	36	80	141.8 (140*)	177.3 (175*)

*Note*: The values of apparent transmissibility and permeability have been obtained previously in our laboratory following the polarographic method.^27^, ^28^, ^29^, ^30^ Johnson & Johnson (jnjvisionpro.com), Bausch & Lomb (bausch.com), Alcon (myalcon.com); (*) permeability and transmissibility values measured by the manufacturer; Barrer = 10^−11^ cm^3^(O_2_)·cm·cm^−2^·s^−1^·mmHg^−1^; hBarrer/cm = 10^−9^ cm^3^(O_2_)·cm^−2^·s^−1^·mmHg^−1^.

The values of apparent transmissibility and permeability have been obtained previously in our laboratory following the polarographic method.^27^, ^28^, ^29^, ^30^ The values of oxygen solubility and diffusion coefficient of the lenses listed are in agreement with Del Castillo et al.^1^

### Physiological parameters considered in the 3‐D model

Table 2 shows the values and units used in the new 3‐D model, including separate corneal layer thickness, oxygen tensions at both anterior and posterior corneal surfaces of the total system, the endothelium/stroma/epithelium/tear/lens/tear under open‐ and closed‐eye conditions and values of maximum oxygen consumption.^15^ A central cornea thickness of 532 μm was considered, distributed as 50 μm for the epithelium, 480 μm for the stroma and 2 μm for the endothelium. The peripheral thickness will depend on the specific power of the contact lens. Finally, the oxygen permeability of each part of the cornea, as well as their diffusivities and solubilities, will change with time^15^, ^16^, ^19^, ^24^, ^25^, ^26^, ^31^, ^32^, ^33^ The permeability of the tears was considered as 93 Barrer or Fatt units, considering that the oxygen diffusion coefficient (*D*) and solubility (*k*) of water at 25°C are 30 × 10^−6^ cm^2^ s^−1^ and 3.1 × 10^−5^ cm^3^(O_2_)·cm^−3^·mmHg^−1^, respectively.^34^ Note that cm^3^(O_2_) refers to the equivalent oxygen quantity measured at standard temperature and pressure conditions (STP) in this work.

**TABLE 2:** Parameters considered when calculating the oxygen tension, flux and the oxygen consumption profiles at the endothelium, epithelium and stroma both on‐axis and in the periphery for open and closed‐eye conditions, considering this new 3‐D model.^11^, ^12^, ^13^, ^15^, ^20^, ^25^

Parameter	Symbol	Value	Units
Partial pressure of oxygen at the posterior corneal surface^12^	p_ac_	24.1	mmHg
Partial pressure of oxygen at the CL surface (open eye)^12^	p_air_	155	mmHg
Partial pressure of oxygen at the CL surface (closed eye)^12^	p_air_	61.5	mmHg
Maximum endothelium oxygen consumption^12^	Q_max,end_	47.78 × 10^−5^	cm^3^(O_2_)·cm^−3^·s^−1^
Maximum stroma oxygen consumption^13^	Q_max,str_	5.75 × 10^−5^	cm^3^(O_2_)·cm^−3^·s^−1^
Maximum epithelium oxygen consumption^13^, ^25^	Q_max,epi_	52.27 × 10^−5^	cm^3^(O_2_)·cm^−3^·s^−1^
Stroma oxygen permeability^20^	(Dk)_str_	29.5	Barrer
Endothelium oxygen permeability^20^	(Dk)_end_	5.3	Barrer
Epithelium oxygen permeability^20^	(Dk)_epi_	18.8	Barrer
Tears (water) oxygen permeability^25^	(Dk)_tear_	93	Barrer
Stroma oxygen diffusion coefficient^13^	D_str_	2.81 × 10^−5^	cm^2^ s^−1^
Endothelium oxygen diffusion coefficient^13^	D_end_	0.496 × 10^−5^	cm^2^ s^−1^
Epithelium oxygen diffusion coefficient^13^	D_epi_	1.767 × 10^−5^	cm^2^ s^−1^
Central cornea thickness^25^	CCT	532	μm
Central epithelium thickness^25^	t_ep_	50	μm
Central stroma thickness^25^	t_str_	480	μm
Central endothelium thickness^25^	t_en_	2	μm
K_m_ (metabolic model parameter)^11^	K_m_	2.2	mmHg
Pre‐Lens tear thickness (PrLTF)^25^	t_lac,in_	15	μm
Post‐Lens tear thickness (PoLTF)^25^	t_lac,ex_	5	μm

Abbreviation: CL, contact lens.

### Theoretical model

In our previous studies, FiPy,^35^ a Python‐based finite volume approach, was used to solve numerically the partial differential equation (PDE) describing the diffusion of oxygen in a model of the cornea–lens system. However, in this work, COMSOL Multiphysics v6.2 (comsol.com) was employed for the numerical model implementation. COMSOL offers predefined physics interfaces for diffusion and reaction, with tailored solvers that enable more accurate and less error‐prone solutions. This enhanced integration allows the introduction of multiple related metabolites such as glucose, carbon dioxide, bicarbonate‐ion, lactate‐ion, water, sodium and chloride ions and hydrogen ion into the model.

To determine the lens shape corresponding to a specific power, the lens maker's equation was used, which relates the focal length of a lens to its material and geometry.^36^ The equation is as follows:

(1) $$P=\frac{1}{f}=\left(n-1\right)\cdot \left(\frac{1}{R_{e}}-\frac{1}{R_{i}}+\frac{n-1}{n\cdot R_{e}\cdot R_{i}}\cdot t_{c}\right)$$

where *P* is the power in dioptres, *f* is the focal length, n is the refractive index of the lens material, *t*_*c*_ is the central thickness of the lens and *R*_*e*_ and *R*_*i*_ are the external and internal radii of the lens, respectively. The internal radius was taken as the external radius of the cornea plus the tear thickness. The external radius for the different powers was found using Equation (1), given the refractive coefficient and central thickness of the material. The central thickness values shown in Table 2 were used for all the negative powered lenses, whereas for positive lenses, the central thickness was calculated to have a thickness in the periphery equal to the central thickness of the same material for a negative power.

**FIGURE 1:**
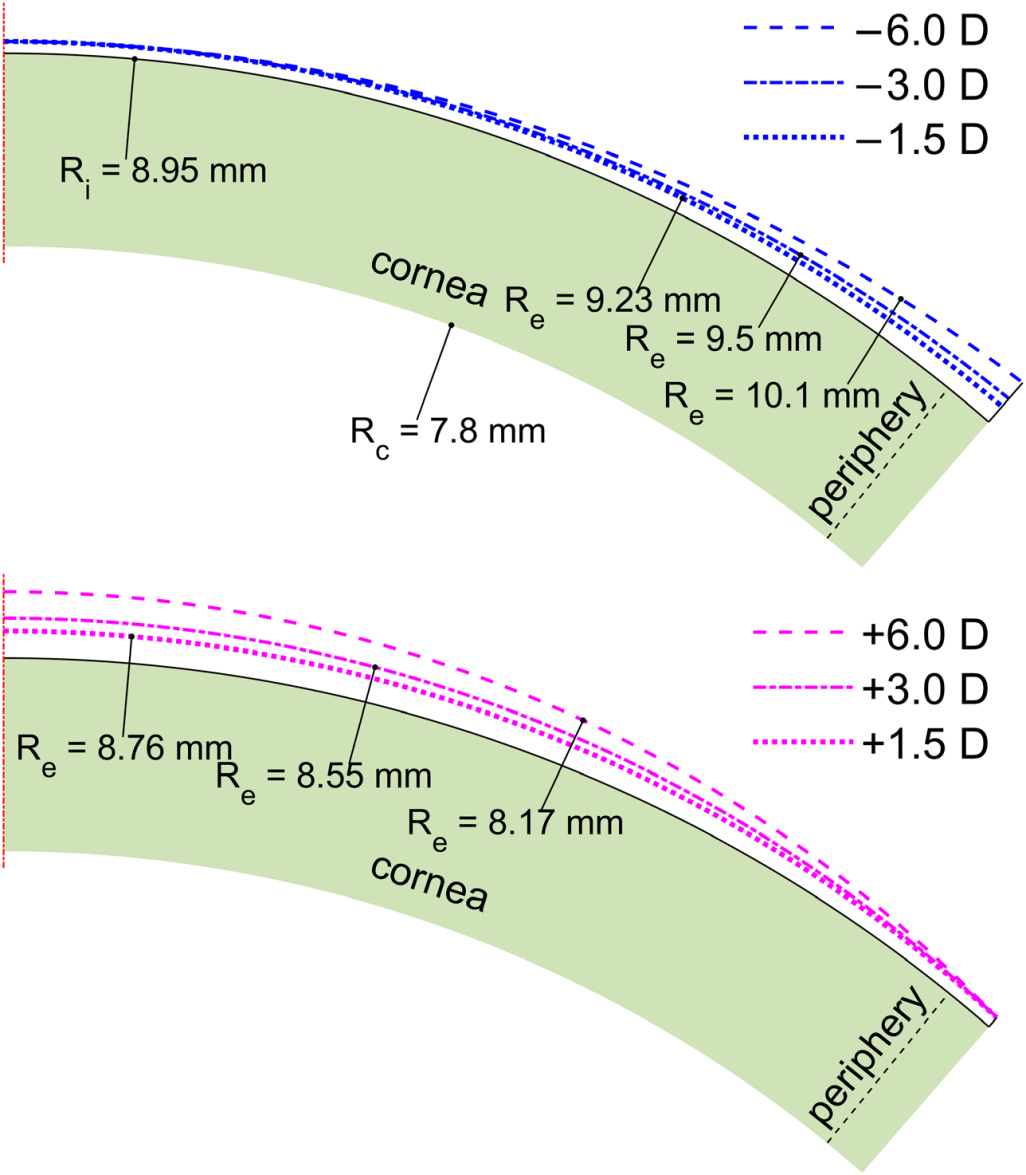
Lens shapes deduced from the power for Galyfilcon A lens material (jnjvisionpro.com). The position of the periphery for transverse analysis is also shown (39° from the axis). The top and bottom figures show the thickness variation for negative and positive lenses, respectively. Re and Ri are the external and internal radii of the lens, respectively.

The refractive index of the lens material corresponding to −3.00 D was used to generate the lens shape for other dioptric powers (+3.00 D and ±6.00 D). For negative powers, the central thickness was maintained as the minimum thickness, while for positive lenses, the minimum thickness was set at the periphery. Geometric information was generated in Matlab® (mathworks.com) and correlations as a function of the power were obtained to implement the position of the origin and peripheral limits in the COMSOL geometry, as shown in Figure 1.

Figure 1 shows the thickness variation of lenses for both negative and positive powers. Close inspection of these figures shows that negative lenses showed a greater increase in thickness in the peripheral zone than positive lenses. In the latter case, the increase in thickness was observed in the axial zone, increasing the thickness as the power of the lens increased. Therefore, it is of interest to carry out a 3‐D study of the flow and consumption of oxygen in the cornea since the oxygen transmissibility of the lens varies depending on the thickness of the lens, which, in turn, is a function of its power. From this new model, it will be possible to appreciate the differences in flow and oxygen consumption compared with a 1‐D analysis.

In this implementation, the dependent variable used was oxygen concentration in each of the following domains: pre‐lens tears, lens, post‐lens tears, epithelium, stroma and endothelium.

Assuming steady‐state conditions without convection, the conservation equation for a component *i*, molecular oxygen in this case, reduces to:

(2) $$\nabla \cdot J_{i}=-Q_{i}$$

being *Q*_*i*_ the oxygen consumption and *J*_*i*_ the oxygen flux given by Fick's law as

(3) $$J_{i}=-D_{i}\nabla c_{i}$$

where *c*_*i*_ is the oxygen concentration in any point of the cornea, which, taking into account Henry's law, can be written as *c*_*i*_ = (*k*·*p*)_*i*_, where *k* and *p* are the oxygen solubility and the oxygen tension at point *i*, respectively. Considering Equations (2) and (3), we obtain:

(4) $$\nabla \cdot \left(D_{i}\nabla c_{i}\right)=Q_{i}$$

It should be noted that solving Equation 4 by considering 3‐D axial symmetry using cylindrical coordinates as the left‐hand term represents a divergence that incorporates additional radial dependence that accounts for the area increase of the elements in the radial direction. In contrast, attempting to model 2‐D diffusion in a section of the eye using Cartesian coordinates, as proposed by Kim et al. in appendix A of their work,^21^ would introduce unnecessary simplifications that may compromise the accuracy of the results.

To solve for the oxygen transport in the system (composed of the endothelium, stroma, epithelium, tear layers and lens), the oxygen consumption is given as a function of the oxygen partial pressure, being zero in the contact lens and tear film regions. We consider that aerobic metabolism in each layer of the corneal system is quantified by the Monod kinetics model,^12^ also named the Michaelis–Menten Model,^20^, ^37^, ^38^ where the relationship between oxygen consumption and oxygen tension is given by:

(5) $$Q_{c,n}\left(p_{c,n}\right)=\frac{Q_{c,\max,n}\cdot p_{c,n}}{K_{m}+p_{c,n}}$$

With $Q_{c,\max,n}$ being the maximum corneal oxygen consumption at domain *n* (*n* = epithelium, stroma or endothelium) when the reaction reaches the steady‐state conditions and $p_{c,n}$ is the partial pressure of oxygen at *n*. Table 2 shows the values of the parameters for the endothelium, stroma and epithelium. In Equations (3) and (4), the solubility (*k*) and the diffusion coefficient (*D*) are considered as a function of the material, taking constant values within each of the different layers of the system (pre‐lens tear, contact lens, post‐lens tear, epithelium, stroma and endothelium). The value of the Monod kinetics constant is *K*_*m*_ = 2.2 mmHg, which was used previously by Chhabra et al.^11^ This value is based on the oxygen consumption from transient post‐contact lens tear‐film oxygen tension using the kinetics model, and assuming the cornea saturates at 90% oxygen consumption when the oxygen tension is 20 mmHg. Using this approach, one can obtain the complete oxygen tension profile, the oxygen flux profile and the oxygen consumption rate (see Figure S1), provided the continuity of the oxygen tension is satisfied in each of the multiple interfaces considered in the corneal system. Dirichlet boundary conditions for oxygen tension were applied at the external interfaces, as shown in Table 2.

The equations were solved in 3‐D domains assuming axial symmetry. The partition conditions between domains were established according to the quotient providing the oxygen solubility between both phases, and the Neumann boundary conditions (flux continuity) were satisfied on each interface.

The stromal calculation domain was meshed with a fine free triangular mesh with a maximum element size of 5 microns. Afterwards, for the other calculation domains (tears, epithelium, endothelium and lens), a mapped meshing was used, as they were much thinner than the stroma. The maximum element size in the transverse direction was 1 micron. The complete mesh consisted of 458,410 domain elements and 8998 boundary elements.

For each material and eye‐opening condition, calculations were performed using parametric continuation to study the effects of power or refractive index. The software's post‐processing capabilities were used to calculate the oxygen flux and oxygen consumption from the oxygen concentration solutions obtained, as well as Equations 4 and 5. In this way, contour plots and flow profiles could be obtained in the central region or the periphery. The software's own integration capacity was used to obtain average values along the interfaces between domains and average oxygen consumption values in specific areas.

## RESULTS

**FIGURE 2:**
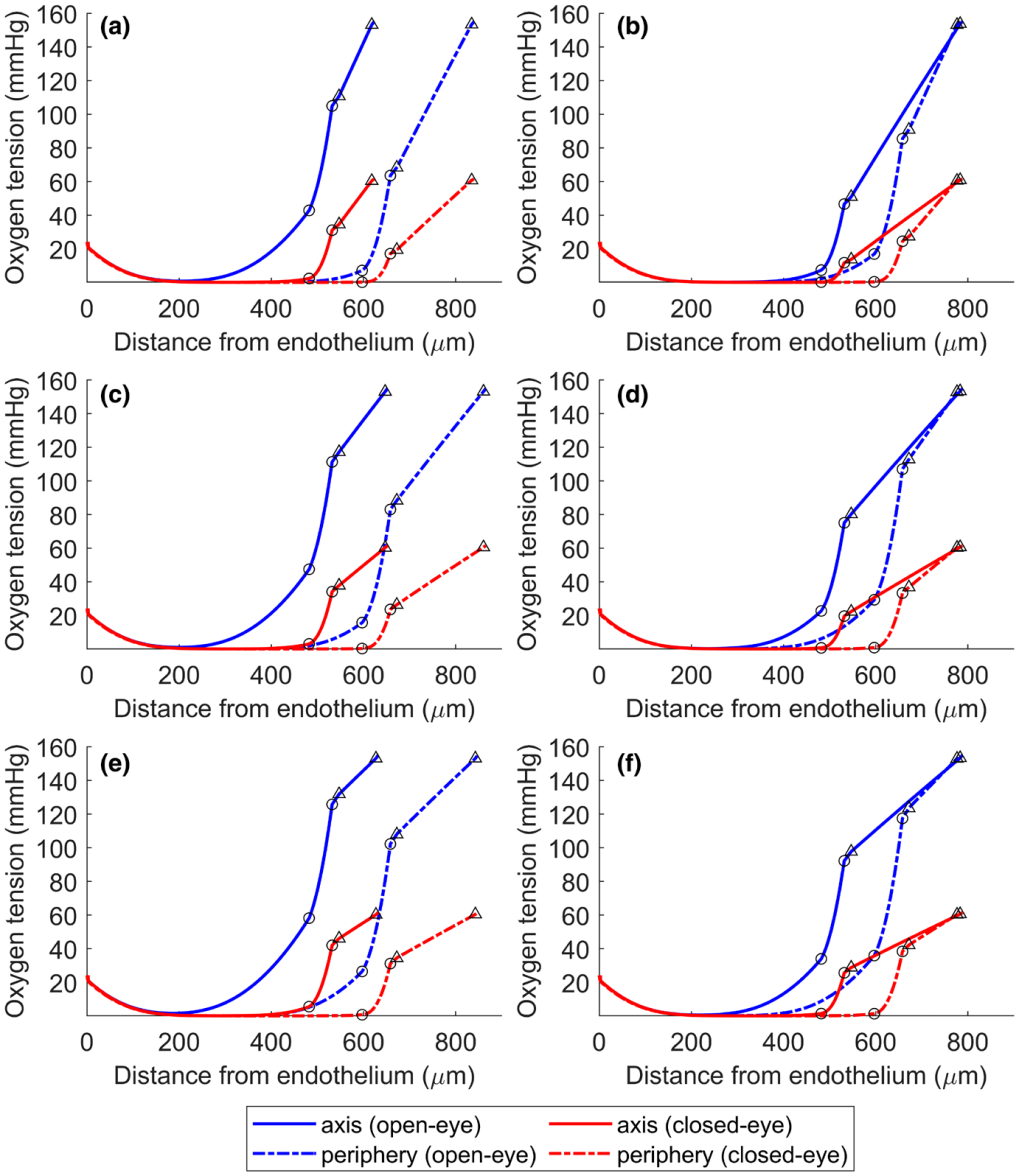
Oxygen tension profiles in the axis and periphery of the eye for the three reference materials and powers of −3.00 D and +3.00 D under open‐eye (blue line) and closed‐eye (red line) conditions. (○ = epithelium, Δ = lens). (a) Galyfilcon A, −3.00 D, (b) Galyfilcon A, +3.00 D, (c) Balafilcon A, −3.00 D, (d) Balafilcon A, +3.00 D, (e) Lotrafilcon A, −3.00 D, (f) Lotrafilcon A, +3.00 D.

Figure 2 shows the oxygen tension profiles for open‐ and closed‐eye conditions for the reference lens materials (Galyfilcon A, Balafilcon A and Lotrafilcon A) for −3.00 D and +3.00 D lenses. The figures include the oxygen tension through the radial axis and peripheral zone. Similar plots were obtained for −6.00 D and +6.00 D lenses (see Figure S2).

The oxygen tension profile was continuous. The main drop in oxygen tension was produced in the region of the CL, especially at the periphery for negative lenses, because of the lower transmissibility of the lens due to the greater thickness in the periphery than at the centre.

Inspection of Figure 2 shows a greater drop in oxygen tension at the periphery than centrally for all lens materials and powers, regardless of whether the eye was open or closed.

Focusing on a lens made from Galyfilcon A with a power of −3.00 D (Figure 2a), a narrow zone with very low oxygen tension (close to 200 μm from the endothelium) was observed along the axis of the eye (solid lines) under open‐eye conditions (blue curves). However, for a +3.00 D lens (Figure 2b), this zone extended up to 400 μm. Considering both powers, the low oxygen concentration zones extended up to 500 μm from the endothelium in the closed‐eye condition because of the low oxygen tension. Comparing the results from the peripheral zone of the eye with the on‐axis findings shows an increased range of the curves; a consequence of the greater thickness of the cornea in the periphery, but also, in all cases, a greater area of the low oxygen tension zone.

If the results obtained for Galyfilcon A are compared with those for Balafilcon A (Figure 2c,d) and Lotrafilcon A (Figure 2e,f) under the same conditions, then one can observe slightly higher levels of oxygen tension as a consequence of the greater oxygen transmissibility of these lenses. These differences are more evident centrally in the case of positive lenses and in the periphery for negative lenses because of the greater thickness in these areas. Notice that for a lens made of Balafilcon A material with a power of −3.00 (Figure 2c), a narrow zone with very low oxygen tension (200 μm from the endothelium) is observed on the axis of the eye under open‐eye conditions. This zone extends up to 490 μm for a +3.00 D Balafilcon A lens and up to 480 μm for a +3.00 D Lotrafilcon A lens (Figure 2d,f). In the case of both powers, the zone of low oxygen concentration extends up to 500 μm from the endothelium in the closed‐eye condition because of the low oxygen tension imposed.

Positive powered lenses are more problematic than negative lenses because they are thicker throughout their profile, as shown in Figure 2b,d,f. This extra thickness leads to lower oxygen tension values in most of the stromal region, especially under closed‐eye conditions.

Comparing the decrease in oxygen tension between the tear/epithelium and epithelium/stroma interfaces for the Galyfilcon A lenses, one can observe a decrease from 105 to 43 mmHg on‐axis and from 64 to 7 mmHg in the periphery, under open‐eye conditions and for a power of −3.00 D. For Balafilcon A lenses, these values varied from 111 to 47 mmHg on‐axis and from 83 to 16 mmHg in the periphery under the same conditions. The model predicts that for Lotrafilcon A lenses, these values should vary from 125 to 58 mmHg on‐axis and from 102 to 27 mmHg in the periphery under open‐eye conditions. Considering the same materials but for a +3.00 D lens, these values will change substantially, with a range of 47–7 mmHg on‐axis and 85–17 mmHg in the periphery for a Galyfilcon A lens, from 75 to 23 mmHg on‐axis and 107 to 29 mmHg at the periphery of a Balafilcon A lens, with on‐axis and peripheral values of 92–34 mmHg and 117–36 mmHg, respectively, for a Lotrafilcon A lens.

Figure 2a compares well with Figure 2 of Aguilella‐Arzo and Compañ,^25^ where the same lens (a −3.00 D Galyfilcon A lens) was studied using different numerical procedures based on FiPy^35^ and a different hypothesis for oxygen consumption in the epithelium. Here, in this new 3‐D model, there is the possibility of inspecting different lens geometries using lenses of additional power.

**FIGURE 3:**
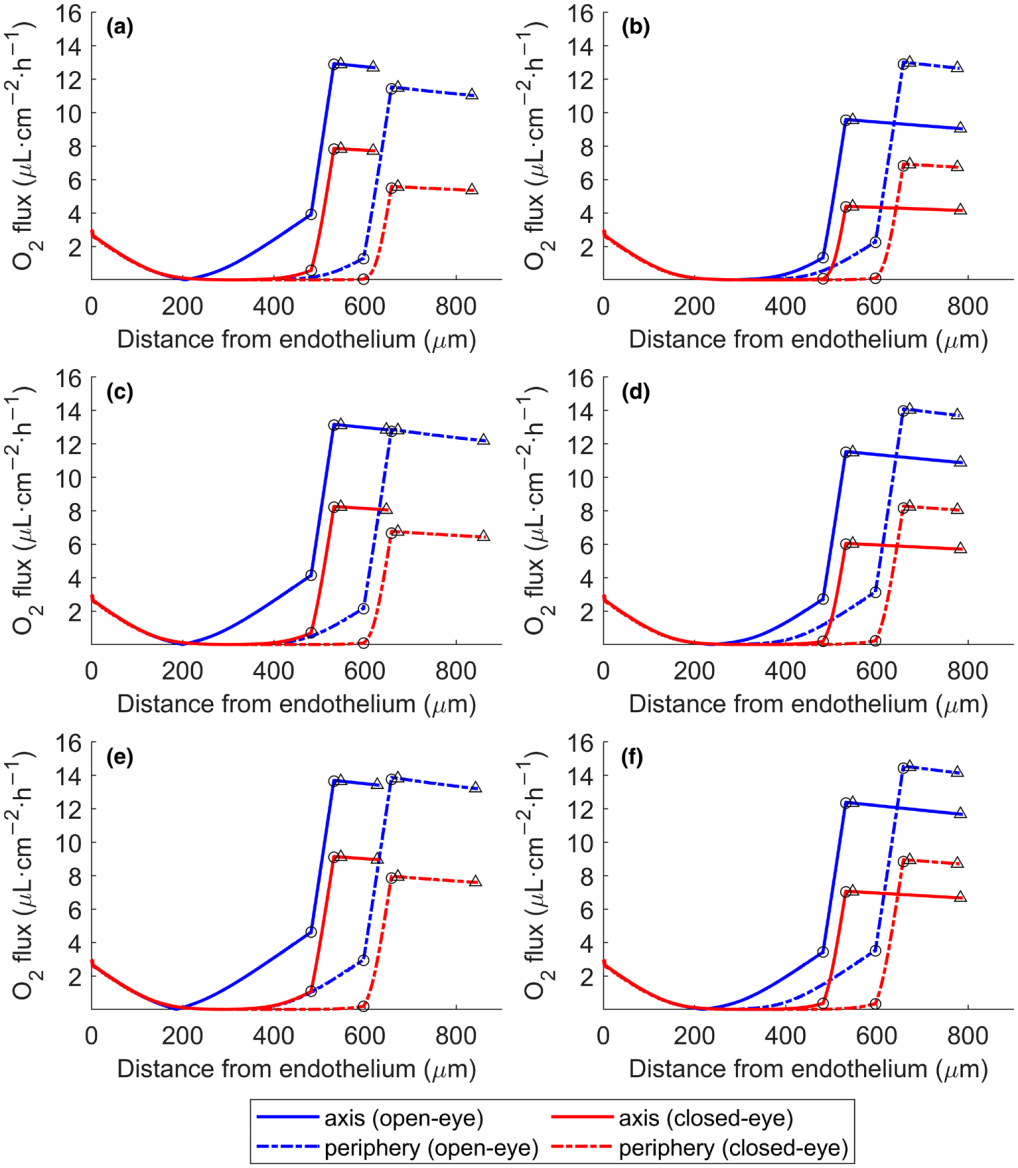
Oxygen flux profiles in the axis and periphery of the eye for the three reference materials and −3.00 D and +3.00 D lens powers in open‐eye (blue line) and closed‐eye (red line) conditions (○ = epithelium, Δ = lens). (a) Galyfilcon A, −3.00 D, (b) Galyfilcon A, +3.00 D, (c) Balafilcon A, −3.00 D, (d) Balafilcon A, +3.00 D, (e) Lotrafilcon A, −3.00 D, (f) Lotrafilcon A, +3.00 D.

Figure 3 depicts the results of the 3‐D model calculations for the magnitude of oxygen flux, under both open‐ and closed‐eye conditions, using the three different lenses with powers of −3.00 D and +3.00 D. The flux magnitude is plotted near the axis and periphery of the eye, represented by the solid and dashed lines, respectively. A similar representation is shown in Figure S3 for −6.00 D and +6.00 D lenses. It can be observed that the varying decrease in the magnitude of oxygen flow, as a consequence of the transition from the open‐ to the closed‐eye condition, depends on the geometry of the lens (related to the power), the location (on‐axis or peripheral) and the lens material.

For the −3.00 D lens (Figure 3a,c,e), the flux was lower near the periphery where the lens was thicker. Conversely, for the +3.00 D lens, the flux was higher near the periphery due to the reduced thickness (Figure 3b,d,f).

Figure 3a reproduces very closely the results of oxygen flux obtained in Figure 2 of Aguilella‐Arzo and Compañ^25^ for the −3.00 D Galyfilcon A lens. However, based on this new model, it is apparent that, for this material, the flux crossing the lens at the periphery was significantly lower than at the axis. This reduction was not observed for the other lens materials having the same power (Figure 3c,e). This can be attributed to the oxygen permeability of the material. The Galyfilcon A material is less oxygen permeable than the others tested here. This information shows that the type of contact lens can cause inflammation of the cornea due to the generation of hypoxia in the epithelium.

Additionally, in Figure 3b, the strong influence of geometry is indicated in a positive Galyfilcon A lens. This shows an approximate 26% reduction in oxygen flux centrally under open‐eye conditions when changing from a Galyfilcon A −3.00 D lens (12.9 μL·cm^−2^·h^−1^) to a +3.00 D lens (9.5 μL·cm^−2^·h^−1^). The effect is reduced near the system's periphery, which shows the complex interplay between the lens geometry and the cornea in determining the oxygen flow and distribution in the cornea–lens system.

In Table 3, for the three lens materials studied, the estimated values of oxygen flux in μL·cm^−2^·h^−1^ exchanged between parts of the cornea (tear/epithelium and epithelium/stroma) analysed from the 3‐D model near the axis A (0°–13.6°), middle ring M (13.6°–27.3°) and peripheral ring P (27.3°–41°) areas are presented. The values of oxygen received by the stroma from the endothelium were almost independent of the lens material (2.61–2.64 μL·cm^−2^·h^−1^ in open‐eye conditions and 2.64–2.67 μL·cm^−2^·h^−1^ in closed‐eye conditions for all endothelium areas and lens materials). For all lenses and materials, an evident reduction in flux can be observed with respect to the no‐lens case as the modulus of the power increases. Furthermore, the relative contribution to the oxygen flux of the A, M and P areas changes with the lens power. For negative powers, the ratio of the flux at A to the flux at P increased, while it decreased for positive lenses.

**TABLE 3:** Oxygen flux (μL·cm^−2^·h^−1^) exchanged from  tears posterior to the lens to epithelium and from epithelium to stroma wearing lenses having different optical power (OP) under open‐eye and closed‐eye conditions and in different areas of the cornea [A: Axis area (0–13.6°), M: Middle ring (13.6–27.3°), P: Peripheral ring (27.3–41°)].

Lens name (*)	Eye	OP	From tears to epithelium			From epithelium to stroma		
			A	M	P	A	M	P
Galyfilcon A	Open	−6	12.74	11.92	10.05	3.64	2.53	0.86
		−3	12.87	12.61	11.89	3.76	3.09	1.81
		+3	9.76	10.53	12.24	1.36	1.52	2.02
		+6	7.33	8.31	10.93	0.39	0.52	1.23
	Closed	−6	7.50	6.27	4.63	0.44	0.17	0.03
		−3	7.69	7.04	5.98	0.49	0.26	0.08
		+3	4.51	5.00	6.28	0.07	0.07	0.09
		+6	3.12	3.63	5.24	0.02	0.03	0.05
Balafilcon A	Open	−6	13.10	12.75	11.84	3.96	3.21	1.77
		−3	13.17	13.15	12.97	4.03	3.56	2.59
		+3	11.69	12.29	13.54	2.74	2.82	3.05
		+6	9.94	10.81	12.76	1.47	1.70	2.42
	Closed	−6	8.04	7.22	5.93	0.59	0.29	0.08
		−3	8.16	7.78	7.05	0.63	0.39	0.15
		+3	6.16	6.64	7.76	0.20	0.20	0.22
		+6	4.63	5.22	6.84	0.07	0.08	0.13
Lotrafilcon A	Open	−6	13.66	13.52	13.07	4.48	3.89	2.67
		−3	13.72	13.80	13.86	4.53	4.14	3.33
		+3	12.51	13.03	14.08	3.44	3.45	3.51
		+6	11.26	11.98	13.54	2.39	2.57	3.04
	Closed	−6	8.99	8.35	7.18	0.96	0.55	0.17
		−3	9.08	8.81	8.21	1.00	0.69	0.30
		+3	7.17	7.59	8.53	0.36	0.35	0.34
		+6	5.72	6.30	7.76	0.15	0.16	0.22
No Lens	Open	0	14.50	14.83	15.41	5.27	5.10	4.73
	Closed	0	10.46	10.62	10.86	1.80	1.58	1.16

*Note*: (*) United States Adopted Name (USAN) for the lens.

The values shown in Table 3 compare very well with those reported previously using a 3‐D model.^25^ The small differences between the two studies can be attributed mostly to the subtle differences in geometry and epithelium oxygen consumption between the investigations, validating the results presented here.

The results obtained in the current work validate previous studies in similar systems but also give a useful perspective of 1‐D models when the 3‐D model is applied to identical lens materials. Close inspection of the work by Compañ et al.,^24^ where a Balafilcon A lens (and others) was studied using a 1‐D diffusion model with an oxygen consumption rate given by the Monod kinetics model, resulted in the conclusion that the on‐axis oxygen flux for a positive lens gave practically the same result. For example, for the −3.00 D Balafilcon A lens, a discrepancy of 9.6% was found between the average oxygen flux at the epithelium/tear interface during open‐eye viewing (13.06 μL·cm^−2^·h^−1^), compared with that obtained in Compañ et al.^24^ However, for the Galyfilcon A lens material, this discrepancy was around 13.5% taking the average values at the epithelium/stroma interface, where the value was ~3.34 μL·cm^−2^·h^−1^, compared with ~3.08 μL·cm^−2^·h^−1^ in this work under open‐eye conditions (a discrepancy of 7.8%). The differences were less apparent under closed‐eye viewing, which makes it difficult to predict the failure of the 1‐D model a priori. All models coincide near the endothelium where the oxygen tension and, consequently, flux and oxygen consumption are strongly determined by the close inner boundary condition. Comparison of the values from the 1‐D model used in Compañ et al.^16^ and the 3‐D model presented here for a −3.00 D Galyfilcon A lens supports the idea that a 1‐D model compares more favourably for the transport of oxygen near the axis, as one would deduce from considerations of symmetry, given that on‐axis the transport has only the axial component in coincidence with the 1‐D transport model. For the 1‐D model, the values were 9.21, 3.46 and 5.09 μL·cm^−2^·h^−1^ at the epithelium/tear, stroma/epithelium and endothelium/stroma, respectively, whereas for the 3‐D model, the respective values were 12.87, 3.94 and 2.67 μL·cm^−2^·h^−1^. Similar discrepancies were observed for the other materials, that is, Balafilcon A and Lotrafilcon A.

Careful observation of the variation in flux shown in Figure 3 (or Figure S3) with lens power for a given material indicates that most of the flux enters perpendicular to the surface of the eye. This is manifested by the approximate constancy of the oxygen flux in the contact lens, except for small variations due to the fact that the external plane of the lens is not exactly concentric to the eye's surface. Furthermore, positive lenses have greater thickness in the axis (radial) versus the peripheral direction, which allows for greater diffusion through the periphery compared with the central area of the lens.

For a negative lens, the on‐axis flux was higher than in the periphery because of the greater lens thickness. In this case, for open‐eye conditions, the oxygen transferred from the epithelium to the stroma was higher than that received by the stroma from the endothelium, up to an angle of about 32° from the axis. For closed‐eye conditions, the oxygen flux received from the tears is substantially decreased, with most of the oxygen being received from the endothelium.

For positive lenses, the situation is reversed because of the greater thickness in the lens centre. Under open‐eye conditions, the stroma can receive more oxygen from the tears and epithelium than from the endothelium at extreme peripheral locations. Under closed‐eye conditions, the oxygen coming from the tears is also decreased substantially, while the oxygen received from the endothelium overwhelmingly predominates.

**FIGURE 4:**
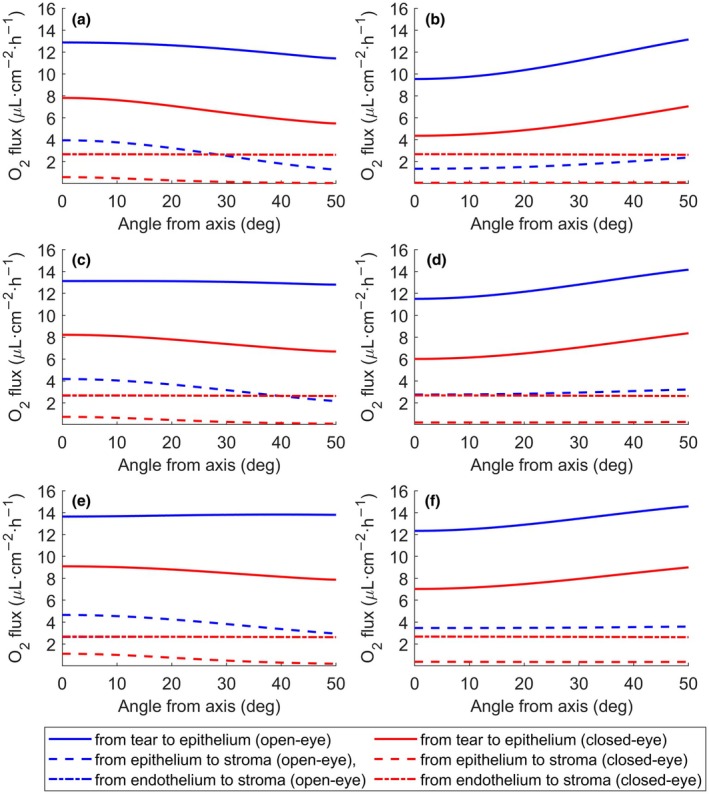
Oxygen flux exchanged between eye layers as a function of the angle from the central axis for the three reference materials and lens powers (−3.00 D and +3.00 D) under open‐eye (blue lines) and closed‐eye (red lines) conditions. (a) Galyfilcon A, −3.00 D, (b) Galyfilcon A, +3.00 D, (c) Balafilcon A, −3.00 D, (d) Balafilcon A, +3.00 D, (e) Lotrafilcon A, −3.00 D, (f) Lotrafilcon A, +3.00 D.

Figure 4 displays the magnitude of oxygen flux crossing the interfaces between domains (from tears posterior to the lens to the epithelium, from epithelium to stroma and from endothelium to stroma) in open‐eye and closed‐eye conditions using the three reference materials (Galyfilcon A, Balafilcon A and Lotrafilcon A lenses) for −3.00 D and +3.00 D lenses. Figure S4 displays the same situation for −6.00 D and +6.00 D lenses. Despite differences in lens materials and geometry, common features emerge. The oxygen flux at the endothelium–stroma interface is nearly independent of the distance from the axis, suggesting that lens geometry has minimal influence on oxygen diffusion in the innermost layer of the cornea.

In contrast, the oxygen flux crossing the epithelium–stroma interface exhibits various behaviours. The crossings corresponding to the almost constant endothelium–stroma and epithelium–stroma flux indicate a reversal of the dominant oxygen flux. Lenses with higher transmissibility and/or lower thickness (as shown in Table 3) show only a slight flux reversal, as in the case of a Lotrafilcon A lens (Figure 6), except under closed‐eye conditions. In the latter case, a consistently lower oxygen flux was observed crossing the epithelium–stroma compared with the endothelium–stroma interface.

Furthermore, a significantly increased oxygen flux was observed, even under closed‐eye conditions, crossing the tear–epithelium interface for all the materials and geometries examined here. This highlights the substantial reduction in flux over the short distance between the tear–epithelium and epithelium–stroma interfaces due to greater oxygen consumption in the epithelium.

**FIGURE 5:**
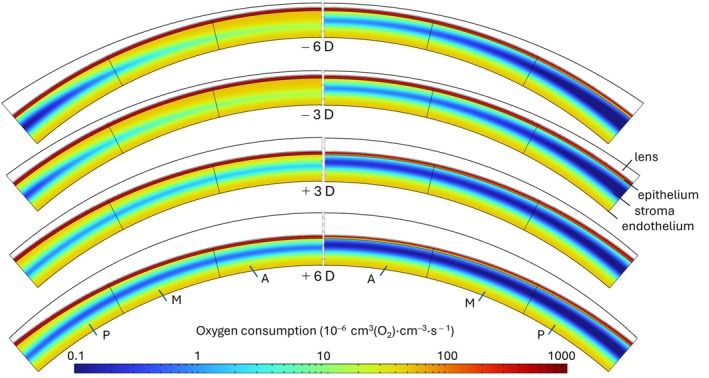
Corneal contour graphs showing oxygen consumption while wearing Galyfilcon A lenses of different powers under open‐eye (left) and closed‐eye conditions (right). From anterior to posterior: Contact lens (white), epithelium (red), stroma (blue) and endothelium (yellow). A, M and P refer to the areas as 0–13.6°, 13.6–27.3° and 27.3–41°, respectively.

**FIGURE 6:**
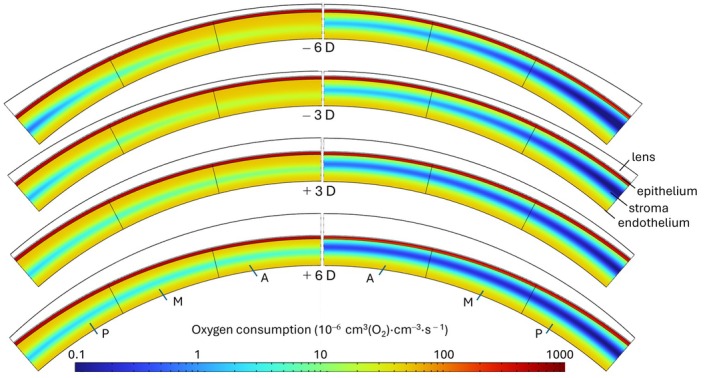
Corneal contour graphs showing oxygen consumption while wearing Lotrafilcon A lenses of different powers in open‐eye (left) and closed‐eye conditions (right). From anterior to posterior: Contact lens (white), epithelium (red), stroma (blue) and endothelium (yellow). A, M and P refer to the areas as 0–13.6°, 13.6–27.3° and 27.3–41°, respectively.

Figures 5 and 6 and Figure S5 display colour maps of oxygen consumption in each layer for the different lens powers and materials. The condition of no contact lens being present is shown in Figure S6.

All figures include both open‐eye (left) and closed‐eye (right) conditions. It may be observed that, in all cases, higher oxygen consumption occurs near the endothelial and epithelial regions. Under closed‐eye conditions, Galyfilcon A lenses exhibited reduced oxygen consumption for all lens powers. Recognising that oxygen consumption is related to the local oxygen concentration, areas of lower consumption correspond with regions of hypoxia, indicating less than ideal material performance under these conditions.

A further interesting observation is the compensating effect of positive lenses on the uneven distribution of oxygen along the cornea. Due to their thinning near the periphery, these lenses compensate for the increased thickness of the cornea. As a result, there is a more homogeneous distribution of oxygen consumption moving away from the central axis, as shown in Figures 5 and 6.

Furthermore, Figures 5 and 6 and Figure S5 provide a clear indication that, for negative powered lenses, although all materials exhibited similar behaviours, a slight advantage was observed with the Lotrafilcon A lenses. This advantage is likely due to their higher oxygen permeability, leading to higher oxygen consumption than for Galyfilcon A lenses.

The sum of the oxygen consumption rates for each point in the epithelium, stroma and endothelium will provide the total corneal oxygen consumption value. These results for open‐ or closed‐eye conditions are shown in Table 4 for different annular areas. The average oxygen consumption in the selected areas (axis, middle ring and periphery of the eye) shows quantitatively important differences caused by the contact lens. The most significant difference was between the axis and the peripheral zones for the +6.00D Galyfilcon A lens under open‐eye conditions, where a relative difference of 54% was observed.

**TABLE 4:** Average oxygen consumption (10^−6^ cm^3^(O_2_)_·_cm^−3^·s^−1^) for different contact lenses, eye conditions and optical powers (OP) in different areas of the epithelium and stroma, as well as a no contact lens condition: Axis area (A, 0–13.6°), middle ring (M, 13.6–27.3°) and peripheral ring (P, 27.3–41°).

Lens name (*)	Eye	OP	Epithelium			Stroma		
			A	M	P	A	M	P
Galyfilcon A	Open	−6	503.5	491.8	436.8	36.3	28.0	16.4
		−3	504.3	499.2	480.1	37.0	31.1	21.4
		+3	463.9	471.4	486.1	22.6	22.2	22.5
		+6	382.3	406.9	460.9	16.7	16.4	18.3
	Closed	−6	389.3	318.8	218.3	17.0	14.4	12.1
		−3	397.2	354.5	280.0	17.3	14.9	12.3
		+3	244.9	257.7	294.1	14.8	13.8	12.4
		+6	170.9	188.1	246.6	14.5	13.6	12.2
Balafilcon A	Open	−6	505.7	500.2	478.9	38.2	31.8	21.2
		−3	506.1	503.3	494.7	38.6	33.8	25.4
		+3	494.9	496.6	500.1	30.9	29.6	27.8
		+6	467.9	476.7	492.5	23.2	23.2	24.5
	Closed	−6	411.0	362.3	277.9	17.9	15.1	12.3
		−3	415.6	385.9	327.7	18.1	15.7	12.7
		+3	328.9	336.7	357.8	15.5	14.6	13.1
		+6	251.2	268.5	318.4	14.8	13.9	12.6
Lotrafilcon A	Open	−6	508.5	505.5	495.5	41.3	35.7	25.9
		−3	508.7	507.0	502.3	41.6	37.1	29.3
		+3	502.0	502.5	503.9	35.1	33.2	30.2
		+6	490.0	493.4	500.0	28.8	28.2	27.8
	Closed	−6	443.1	407.9	332.9	20.1	16.6	12.8
		−3	445.9	424.6	375.6	20.4	17.4	13.5
		+3	375.5	378.7	388.6	16.5	15.4	13.7
		+6	307.0	320.8	357.7	15.2	14.3	13.1
No lens	Open	0	511.7	511.2	510.3	45.8	42.5	36.6
	Closed	0	478.0	473.2	461.0	25.3	22.5	18.0

*Note*: (*) United States Adopted Name (USAN) for the lens.

For the endothelium, the values obtained in the areas of the axis, middle and peripheral rings were practically the same (4.353 × 10^−4^, 4.351 × 10^−4^ and 4.348 × 10^−4^ cm^3^(O_2_)·cm^−3^·s^−1^, respectively) for all powers, materials and eye conditions studied. Accordingly, these values are not included in Table 4.

For all lenses and materials, an evident reduction in oxygen consumption can be observed with respect to the no‐lens situation as the modulus of the power increased. For the no‐lens case, the oxygen consumption was similar in all areas (A, M and P). The use of negative lenses led to a decrease in oxygen consumption, predominantly in the periphery, while positive lenses caused significantly decreased oxygen consumption in all areas.

**FIGURE 7:**
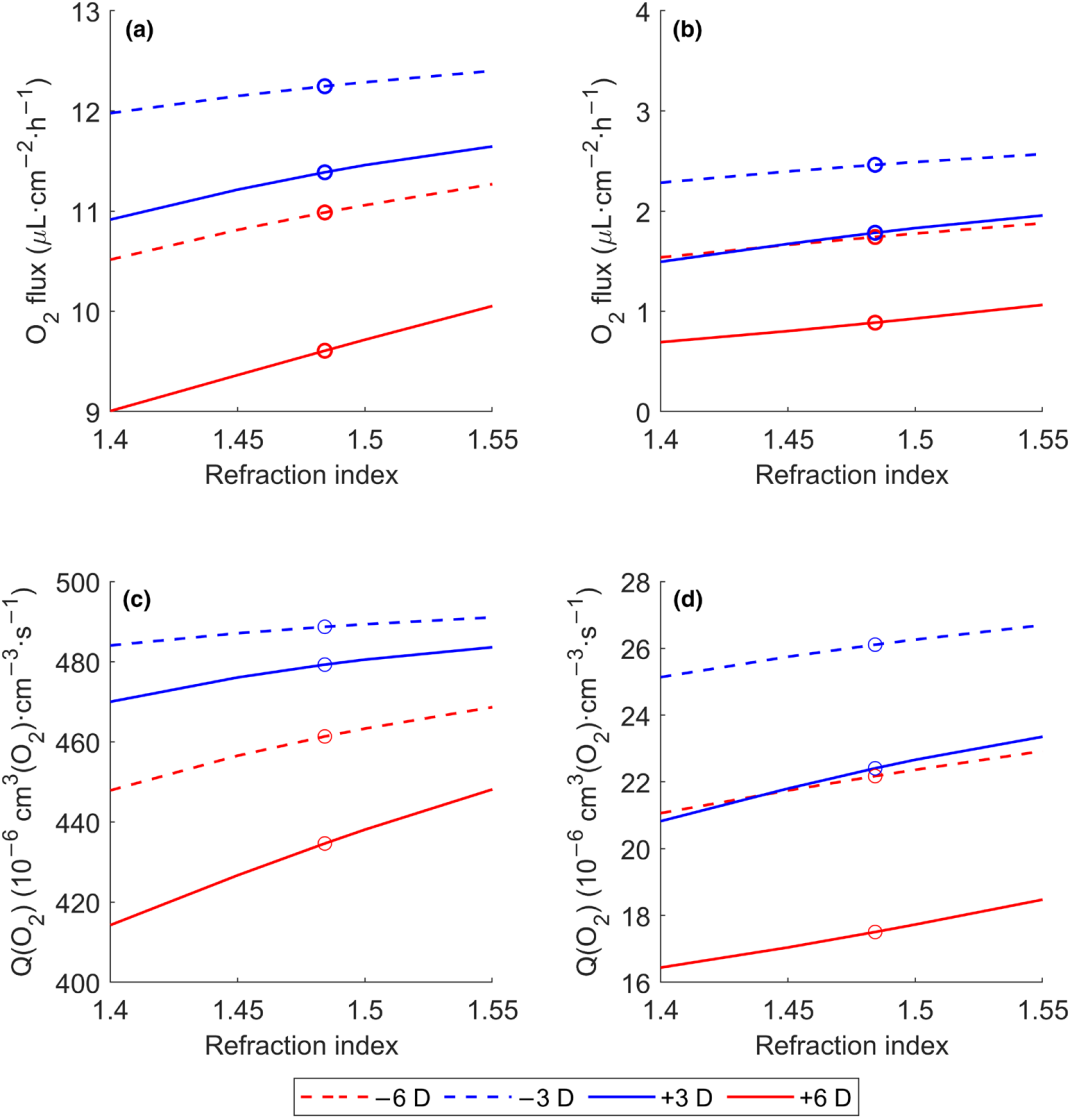
Parametric study of the effect of the refractive index on the average oxygen flux and oxygen consumption wearing Galyfilcon A lenses of varying powers under open‐eye conditions: (a) oxygen flux exchanged from tears to the epithelium; (b) oxygen flux exchanged from the epithelium to the stroma; (c) oxygen consumption in the epithelium; (d) oxygen consumption in stroma; (o) values at the refractive index measured and used in the remainder of the study (1.4842).

Finally, the influence of the lens refractive index was investigated. Figure 7 shows a parametric study of the refractive index across its usual range for the Galafylcon A lens at the powers considered in this study. Across all powers, the higher the refractive index, the lower the lens thickness. In the case of negative lenses, a higher refractive index meant less thickness in the periphery and conversely, in the case of a positive lenses, less thickness in the centre. This leads, in general, to an increase in oxygen flow to the stroma and consequently, an increase in oxygen consumption in both the epithelium and the stroma.

For example, taking the results shown in Figure 7 for a −6.00 D lens, an increase in the refractive index from 1.4842 to 1.5500 (4.43%) yielded an increase in oxygen flux of 2.57% to the epithelium and 7.89% to the stroma. This increased oxygen flux caused an increase in the oxygen consumption rate of 1.59% and 3.38% in the epithelium and stroma, respectively. These variations are more significant for a +6.00 D lens, where for the same variation in refractive index, oxygen flux to the epithelium and stroma increased by 4.65% and 19.98%, respectively, while oxygen consumption increased 3.11% in the epithelium and 5.54% in the stroma. The increments are less significant for lenses of lesser power, such as ±3.00 D.

## DISCUSSION

It is well established that a decrease in oxygen tension results in reduced oxygen flow and consumption, prompting a shift in corneal physiology towards anaerobic metabolism, thereby increasing lactate production and leading to corneal swelling.^39^ The 3‐D modelling presented here predicts that under open‐eye conditions, the oxygen tension profiles and flux for specific anterior corneal surface P_O2_ values exceed 60 mmHg across all of the lens materials studied, both centrally and peripherally. This suggests that these lenses, ranging in powers from +3.00 to −3.00 D, provide higher oxygen tension values to the cornea compared with the closed‐eye environment, where the palpebral conjunctiva typically achieves a value of around 60 mmHg.^5^, ^8^, ^15^

Through a combination of modelling and experimentation, a critical oxygen tension (COT) ranging from 70 to 125 mmHg has been proposed as the threshold below which corneal physiology becomes compromised, leading to corneal oedema and other associated metrics.^40^, ^41^, ^42^, ^43^, ^44^ This new 3‐D model yields average oxygen flux values into the corneal tear/epithelium interface for a −3.00 D lens of 12.25 μL·cm^−2^·h^−1^ for Galyfilcon A, 13.06 μL·cm^−2^·h^−1^ for Balafilcon A and 13.83 μL·cm^−2^·h^−1^ for Lotrafilcon A. Conversely, for a power of +3.00 D, these values are predicted to be 11.38, 12.91 and 13.54 μL·cm^−2^·h^−1^ for Galyfilcon A, Balafilcon A and Lotrafilcon A lenses, respectively, under open‐eye conditions at sea level. Notably, these results align more closely with Takatory et al.^19^ than the previous findings of Jauregui and Fatt.^32^

Examination of Figures 2, 3, 4 indicates that all of the lenses studied predicted average oxygen flux values (both centrally and peripherally) that decreased towards the corneal tear interface, with values of P_O2_ above 70 mmHg at the interface between the epithelium and the post‐contact lens tear layer for negative lenses (−3.00 D) and very close to this value for positive powers (+3.00 D). This oxygen tension value (≥70 mmHg) is comparable with or slightly higher than that observed under the closed eyelid condition.^45^ With these average oxygen flux and oxygen tension values at the epithelium/tear interface, we postulate that the epithelial layer would be adequately supported to maintain optimal physiological conditions when lenses whose transmissibility provides an oxygen tension at the cornea/tear/lens interface ≥70 mmHg. This value could be the critical oxygen tension at the corneal surface. Therefore, contact lenses whose transmissibility creates oxygen tension below this value could induce hypoxic complications such as corneal swelling, loss of transparency, acidosis, limbal hyperaemia or epithelial punctate staining, among others.

Comparison of the values in Table 4 with previous models highlights the limitations of 1‐D models^24^ for depicting the oxygen transport mechanisms in corneal lenses accurately. One of the primary shortcomings of 1‐D models, such as the one described by Compañ et al.,^24^ lies in their inability to account for intricate details such as refractive power. These models are restricted to incorporating lens thickness as the sole geometric characteristic of the lens, which can significantly impair their ability to replicate the intricate oxygen transport dynamics within the corneal tissue. Nevertheless, despite this significant limitation, the results obtained from 1‐D models generally align well with those obtained from more sophisticated 3‐D models. The most notable discrepancy arises in the overestimation of oxygen consumption rates by 1‐D models. For instance, for a −3.00 D Balafilcon A lens under open‐eye conditions, the 1‐D model^24^ predicts oxygen consumption rates of 5.85 × 10^−4^ cm^3^(O_2_)·cm^−3^·s^−1^ in the epithelium and 4.9 × 10^−5^ cm^3^(O_2_)·cm^−3^·s^−1^ in the stroma. In contrast, the current 3‐D model predicts significantly lower oxygen consumption rates of 4.99 × 10^−4^ cm^3^(O_2_)·cm^−3^·s^−1^ in the epithelium and 2.95 × 10^−5^ cm^3^(O_2_)·cm^−3^·s^−1^ in the stroma. Similar discrepancies are observed for closed‐eye conditions, with the 1‐D model predicting consumption rates of 3.55 × 10^−4^ cm^3^(O_2_)·cm^−3^·s^−1^ and 2.8 × 10^−5^ cm^3^(O_2_)·cm^−3^·s^−1^ in the epithelium and stroma, respectively, compared with the 3‐D model predictions of 3.55 × 10^−4^ cm^3^(O_2_)·cm^−3^·s^−1^ and 1.42 × 10^−5^ cm^3^(O_2_)·cm^−3^·s^−1^, respectively.

The subtleties present in the use of lenses having different refractive powers, which can lead to changes in oxygen consumption of more than 100% (see Table 4 for the corneal epithelium of an eye wearing a ±6.00 D Galyfilcon A lenses), can only be explored correctly with a model such as the one presented in this work. These results could not have been obtained accurately for ±6.00 D lenses using a 1‐D model.

Finally, the results for a −3.00 D lens estimate an average oxygen consumption rate at the epithelium around 4.89 × 10^−4^, 4.99 × 10^−4^ and 5.05 × 10^−4^ cm^3^(O_2_)·cm^−3^·s^−1^, for Galyfilcon A, Balafilcon A and Lotrafilcon A lenses, respectively, working under open‐eye conditions and 3.16 × 10^−4^, 3.55 × 10^−4^ and 3.99 × 10^−4^ cm^3^(O_2_)·cm^−3^·s^−1^, for Galyfilcon A, Balafilcon A and Lotrafilcon A, respectively, working under closed‐eye conditions. These results are higher than the values used by Alvord et al.^20^ in his 1‐D model based on the 2005 Brennan Eight Layer (BEL) model,^2^ which used a value of 2.59 × 10^−4^ cm^3^(O_2_)·cm^−3^·s^−1^.

It is important to emphasise that all of the findings presented in this study rely on the application of the nonlinear Monod Kinetics model, which posits that corneal oxygen consumption is a result of aerobic metabolism influenced by oxygen tension, as described in Equation (5) of Chhabra et al.^11^, ^12^ Other researchers have proposed a similar equation but with a different value for the *K*_*m*_ parameter, substituting 0.5 for the value of 2.2 used by Chhabra. This discrepancy in *K*_*m*_ values stems from consideration of mitochondrial activity in cell respiration under hypoxic conditions.^46^ Under this premise, the minimum observed oxygen flux into the stroma (Figure 3) and oxygen tension (Figure 2) would shift towards that of the aqueous humour (data not shown).

Lastly, if an increase in the refractive index of 4.43% from the reference case value of 1.4842 is considered, then it can be seen that the higher the power modulus, the greater the improvement in oxygen flux, with increases in the flux to the epithelium of 1.25% and 2.57% for −3.00 D and −6.00 D lenses, respectively. This effect is even greater in the case of positive lenses, where a 2.27% and 4.65% improvement was obtained for a +3.00 D and +6.00 D lens, respectively. These results, together with the thickness and geometry of the lens, should be considered in the design of contact lenses for prolonged use, particularly in the case of positive lenses.

## CONCLUSIONS

This study aimed to enhance prior investigations by our group, focusing on a 3‐D treatment with axial symmetry in the context of corneal oxygen dynamics. Our previous work concentrated on the corneal epithelium, acknowledging its maximal oxygen consumption rate as a function of corneal oxygen tension at the cornea/tear/lens interface. Here, we present a more rigorous and comprehensive treatment of diffusion at the cornea–tear–lens system, considering lenses with different oxygen permeability and refractive power.

This approach involved solving 3‐D diffusion equations (with axial symmetry) and integrating the possibility of oxygen influx from the atmosphere through the peripheral cornea and the aqueous humour. The model can involve contact lenses of varying powers, facilitating the modelling of arbitrary lenses under both open‐ and closed‐eye conditions using minimal input values. Numerical solutions of the transport equations yield diverse predictions, including oxygen pressure profiles, flows, consumption rates and integrated values across different corneal layers (i.e., the epithelium, stroma and endothelium).

Importantly, this novel model calculates oxygen tension profiles from the central‐to‐peripheral cornea, allowing for the quantification of metabolite transport and the assessment of corneal oedema. This allows evaluation of the impact of metabolite support from the vascularised limbus and the heightened metabolic demand of the mid‐peripheral and peripheral cornea during soft contact lens wear on corneal oedema. Notably, existing models by Alvord^20^ and Takatori‐Radke^19^ lack these capabilities.

By incorporating limbal metabolic support and considering various transport mechanisms, this model provides a valuable tool for predicting the oxygenation safety of contact lenses across the entire cornea. Furthermore, the model can be adapted easily to calculate steady‐state and transient profiles of other metabolites, such as glucose, lactate, bicarbonate, hydrogen, sodium and chloride, amongst others. We anticipate that these enhancements will contribute to a deeper understanding of the interplay between metabolic reactions and oxygen transport, paving the way for future studies in this domain.

The decrease in oxygen consumption leads to a physiological shift towards anaerobic metabolism, resulting in elevated lactate production and consequent corneal swelling. Through a combination of computational models and experimental investigations, we postulate the existence of a ‘critical oxygen tension’ specific to the anterior cornea, below which corneal physiology becomes compromised, leading to oedema. An oxygen tension between 70 and 125 mmHg at the cornea/contact lens interface is necessary to avoid hypoxic complications such as corneal swelling, loss of transparency, corneal oedema, corneal stroma acidosis, epithelial punctate staining, limbal hyperaemia and endothelial polymegathism.

Thus, aerobic metabolism is sustained at the cornea–tear interface (anterior corneal surface) for oxygen partial pressures (P_O2_) ranging from approximately 70–125 mmHg. This phenomenon holds true across all the contact lens types studied in this work under open‐eye conditions. Conversely, aerobic metabolism is not maintained adequately under closed‐eye conditions, predisposing the cornea to anaerobic metabolism and subsequent oedema upon prolonged exposure to such conditions.

With this model, in addition to calculating the oxygen flux, oxygen consumption and pressure profiles in each part of the cornea can easily be modified to calculate steady‐state and transitory profiles of other kinds of reactive metabolites involved in corneal metabolism as glucose or ions, such as lactate, bicarbonate, hydrogen, sodium and chloride, among others. This could help to understand the connection between metabolic reactions and oxygen transport, which will be studied further in future work.

## AUTHOR CONTRIBUTIONS

**José M. Gozálvez‐Zafrilla:** Conceptualization (equal); data curation (equal); formal analysis (equal); investigation (equal); methodology (equal); software (equal); supervision (equal); validation (equal); visualization (equal); writing – review and editing (equal). **Marcel Aguilella‐Arzo:** Conceptualization (equal); formal analysis (equal); investigation (equal); methodology (equal); software (equal); supervision (equal); visualization (equal); writing – original draft (equal). **Vicente Compañ:** Conceptualization (equal); formal analysis (equal); investigation (equal); methodology (equal); project administration (equal); resources (equal); supervision (equal); validation (equal); visualization (equal); writing – original draft (equal); writing – review and editing (equal).

## FUNDING INFORMATION

None.

## CONFLICT OF INTEREST STATEMENT

The author declares no conflicts of interest.

## Supporting information


Figure S1.
