# Supplementary material for: A new three‐dimensional model to describe human corneal oxygenation during contact lenses wear
Source: Ophthalmic Physiol Opt. 2025 Apr 19;45(5):1126–41. doi: 10.1111/opo.13510 (PMC12153035; doi:10.1111/opo.13510)
Supplement: Supplementary file 1 — Figure S1. [file OPO-45-1126-s001.docx]

**Supplementary Information**

**A new 3-dimensional model to describe human corneal oxygenation during contact lenses of different optical power wear.**

**José M. Gozálvez-Zafrilla^1^, Marcel Aguilella-Arzo^2^, Vicente Compañ^3,*^**

^1^Institute for Industrial, Radiophysical and Environmental Safety (ISIRYM). Departamento de Ingeniería Química y Nuclear. Universitat Politécnica de Valencia, Campus de Vera s/n, 46020 Valencia, (Spain)

^2^ Departamento de Física. Universitat Jaume I. 12080, Castellón (Spain)

^3,*^ Departamento de Termodinámica Aplicada. Escuela Técnica Superior de Ingenieros Industriales (ETSII). Universitat Politécnica de Valencia, Campus de Vera s/n, 46020 Valencia, (Spain).

(*) The whom correspondence to Vicente Compañ. E-mail: vicommo@ter.upv.es

Figures S1, S2, S3, S4, S5, S6

Figure S1. Oxygen consumption as a function of oxygen tension in the different parts of the cornea wearing a Galyfilcon A lens (Monod model parameters of Table 2)

Figure S2. Oxygen tension profiles in the axis and periphery of the eye for the three reference materials and optical powers −6.00 D and +6.00 D under open-eye (blue line) and closed-eye (red line) conditions (○ = epithelium ends, Δ = lens ends).

Figure S3. Oxygen flux profiles in the axis and periphery of the eye for the three reference materials and optical powers −6.00 D and +6.00 D under open-eye (blue line) and closed-eye (red line) conditions (○ = epithelium ends, Δ = lens ends).

Figure S4. Oxygen flux exchanged between eye layers as a function of the angle taken from the eye axis for the three reference materials and optical powers −6.00 D and +6.00 D in open-eye (blue line) and closed-eye (red line) conditions.


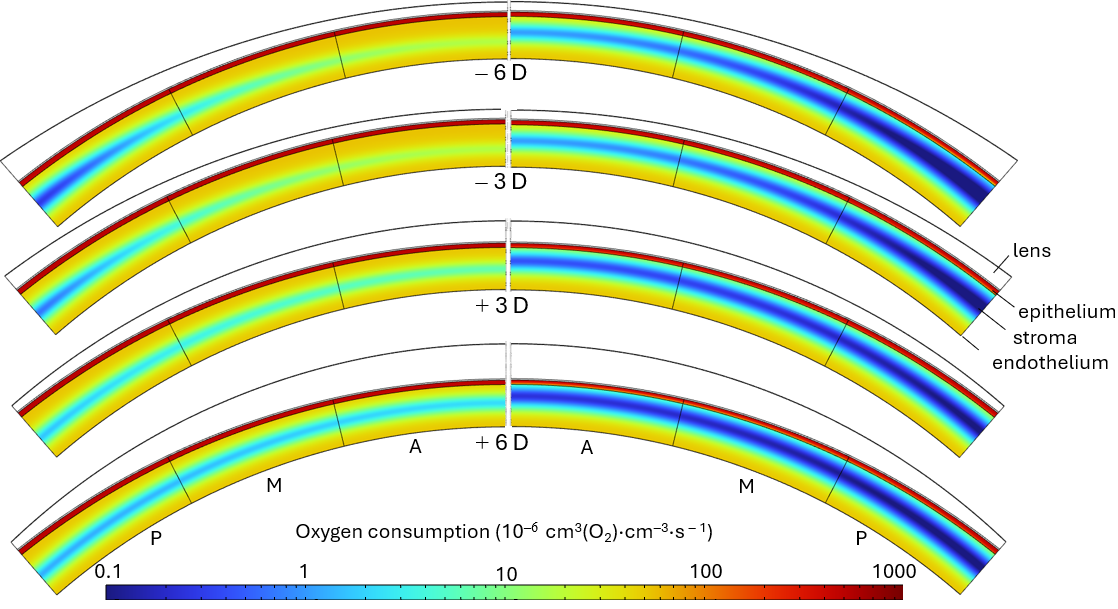


Figure S5. Corneal contour graphs of oxygen consumption wearing Balafilcon A lenses of different diopters in open-eye (left) and closed-eye condition (right) [A: axis area (0-13.6º), M: middle ring (13.6º-27.3º), P: peripheral ring (27.3º-41º)].


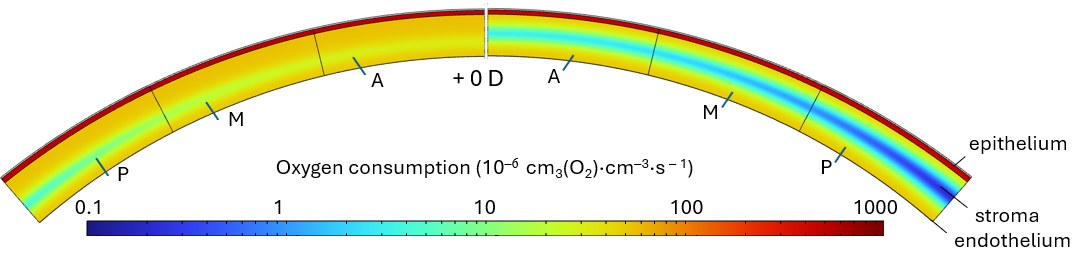


Figure S6. Corneal contour graphs of oxygen consumption without lens in open-eye (left) and closed-eye condition (right). [A: axis area (0-13.6º), M: middle ring (13.6º-27.3º), P: peripheral ring (27.3º-41º)].
